# Amorphous Calcium Phosphate and Amorphous Calcium Phosphate Carboxylate: Synthesis and Characterization

**DOI:** 10.1021/acsomega.3c00796

**Published:** 2023-07-17

**Authors:** Abhishek Indurkar, Rajan Choudhary, Kristaps Rubenis, Mansingraj Nimbalkar, Anatolijs Sarakovskis, Aldo R. Boccaccini, Janis Locs

**Affiliations:** † Rudolfs Cimdins Riga Biomaterials Innovations and Development Centre of RTU, Institute of General Chemical Engineering, Faculty of Materials Science and Applied Chemistry, Riga Technical University, Pulka Street 3, LV-1007 Riga, Latvia; ‡ Baltic Biomaterials Centre of Excellence, Headquarters at Riga Technical University, Kipsalas Street 6A, LV-1048 Riga, Latvia; § Department of Botany, Shivaji University, Kolhapur 416004, Maharashtra, India; ∥ Institute of Solid State Physics, University of Latvia, 8 Kengaraga Str., LV-1063 Riga, Latvia; ⊥ Institute of Biomaterials, Department of Material Science and Engineering, University of Erlangen-Nuremberg, 91085 Erlangen, Germany

## Abstract

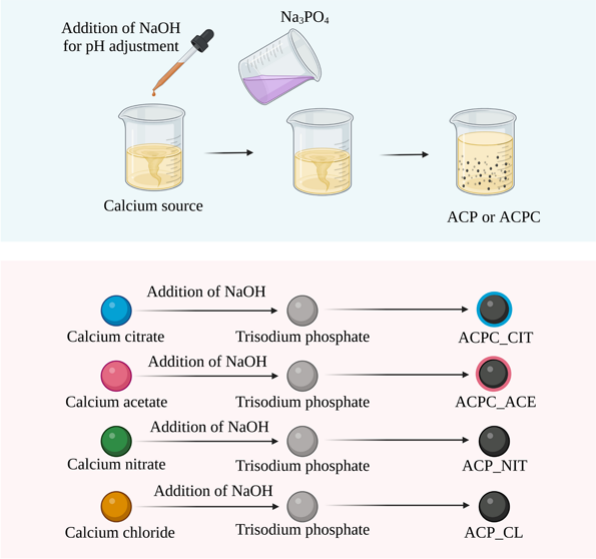

Amorphous calcium phosphate (ACP) is the first solid phase precipitated from a supersaturated calcium phosphate solution. Naturally, ACP is formed during the initial stages of biomineralization and stabilized by an organic compound. Carboxylic groups containing organic compounds are known to regulate the nucleation and crystallization of hydroxyapatite. Therefore, from a biomimetic point of view, the synthesis of carboxylate ions containing ACP (ACPC) is valuable. Usually, ACP is synthesized with fewer steps than ACPC. The precipitation reaction of ACP is rapid and influenced by pH, temperature, precursor concentration, stirring conditions, and reaction time. Due to phosphates triprotic nature, controlling pH in a multistep approach becomes tedious. Here, we developed a new ACP and ACPC synthesis approach and thoroughly characterized the obtained materials. Results from vibration spectroscopy, nuclear magnetic resonance (NMR), X-ray photoelectron spectroscopy (XPS), true density, specific surface area, and ion release studies have shown a difference in the physiochemical properties of the ACP and ACPC. Additionally, the effect of a carboxylic ion type on the physiochemical properties of ACPC was characterized. All of the ACPs and ACPCs were synthesized in sterile conditions, and in vitro analysis was performed using MC-3T3E1 cells, revealing the cytocompatibility of the synthesized ACPs and ACPCs, of which the ACPC synthesized with citrate showed the highest cell viability.

## 1 Introduction

ACP is the first solid phase precipitated from a supersaturated calcium phosphate solution.^1^ Naturally, ACP is synthesized and stabilized by an organic compound (termed the “Howard factor”) in mitochondria of cells.^2^ It is known that the carboxyl group-containing organic compound provides a nucleation site, and the hydrocarbon chain provides an orientation to hydroxyapatite.^3^ Synthetic precipitation reactions of ACP are rapid and highly influenced by the temperature, pH, and concentration of calcium and phosphate precursors.^4^ Due to the triprotic nature of phosphates, variation in pH alters the relative concentration of four protonated forms of phosphoric acid such as H_3_PO_4_ (phosphoric acid), H_2_PO_4_^–^ (dihydrogen phosphate), HPO_4_^2–^ (hydrogen phosphate), and PO_4_^3–^ (phosphates). This leads to variations in chemical composition and the amount of synthesized ACP, thus resulting in difficulties in controlling the formation of ACP.^5^

The interaction of different organic groups with inorganic materials has been a focus of biomineralization and biomaterials research. The role of macromolecules such as collagen, protein, and polymers in nucleation, crystallization, aggregation, and phase transformation of different calcium phosphates in biomineralization has been of particular interest for the last two decades.^6^ Compared to noncollagenous proteins, small organic molecules such as citrate provide more carboxylic for calcium binding.^7^ However, less consideration has been given to incorporating COO^–^-containing small organic molecules in calcium phosphate (CaP), although their role was indicated in numerous postulated biomineralization.^8^ ACP and octa-calcium phosphate (OCP) are precursor phases that gradually crystallize to apatite.^9^ Therefore, crystal growth, nucleation, and stabilization of apatite may be regulated by incorporating COO^–^ in ACP or OCP.^10^ Usually, OCP is synthesized by hydrolysis of tricalcium phosphate (TCP), where the reaction is time-consuming and pH variations are controllable. The reaction parameter permits the addition of carboxylic compounds in developing octa-calcium phosphate carboxylate (OCPC).^11^ In this manner, a series of COO^–^-containing small organic molecules were successfully integrated into OCPC.^12^ On the contrary, ACP was synthesized by a precipitation reaction, and the direct addition of carboxylic acids leads to pH fluctuations affecting the physiochemical properties of the final product.^13^ Due to the synthesis limitations of ACP, less attention has been paid to the development of amorphous calcium phosphate carboxylate (ACPC).

The development of ACPC is a favorable option from the biomimetic point of view. For instance, citrate is a tricarboxylic compound synthesized in mitochondria and present in bone and teeth.^14^ Recent nuclear magnetic resonance (NMR) studies have revealed the presence of citrate bridges between the mineral platelets of bone.^15,16^ Moreover, one-sixth of the available surface area of apatite is covered by citrate.^15,17^ The adsorbed citrate on the surface of apatite offers more COO^–^ groups for collagen binding compared to noncollagenous proteins. The bonded citrate on CaP reduces the hydrophilicity of the surface, making it favorable for binding with nonpolar amino acids such as alanine and proline in the collagen matrix.^18^ In this study, citrate was utilized to develop one of the ACPCs. The literature shows that the OCPC possesses different properties depending upon the type of the incorporated COO^–^ ion.^19^ Unfortunately, such data are not available for carboxylate ions containing ACP. To investigate the effect of carboxylic ion type on the properties of ACPC, we also utilized acetate as a source of the mono-carboxyl compound for the synthesis of ACPC.

**Figure 1:**
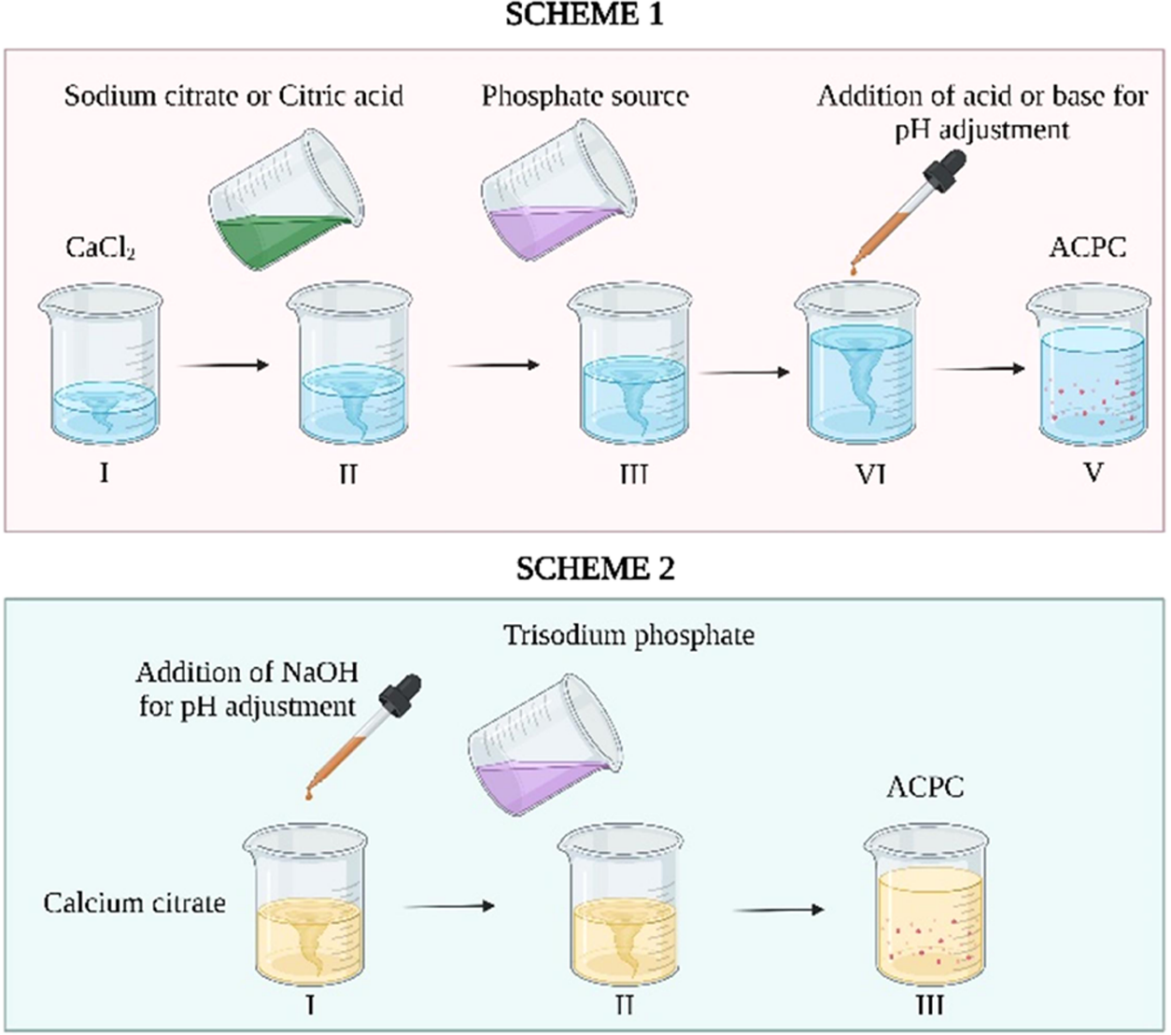
Synthesis mechanism of ACPC. Scheme 1 represents the traditional synthesis approach in which citrate is used as an acid or a base.^20−26^ The reaction of ACPC synthesis is rapid, and due to the triprotic nature of phosphate, fluctuation in pH disturbs the protonated forms of phosphates, which can affect the final product. Scheme 2 represents our synthesis approach in which citrate was used as a calcium salt, and pH adjustments were made before mixing of calcium and phosphate sources. Therefore, the pH is maintained during the reaction.

In the scientific literature, the synthesis of ACP is usually described as a simple one-step process;^4^ on the contrary, ACPC is synthesized by a multistep process.^20−26^ As shown in Figure 1, Scheme 1, the precursor of COO^–^-containing small organic molecules is either used in the acidic or basic form, which can lead to variation in pH, making the reaction tedious, expensive, and affecting the final product. Moreover, ACP/ACPC reactions are rapidly performed at alkaline pH. Therefore, the addition of small organic molecules in the reaction leads to pH fluctuation affecting the reaction’s phosphate species, which can influence the final product. We have developed a simplified one-step synthesis of ACPC, as shown in Scheme 2. This simple approach utilizes calcium salt of COO^–^-containing small organic molecules, resulting in marginal pH variations, and provides a rapid and cost-effective approach. The approach developed was used to synthesize ACPC from calcium citrate (ACPC_CIT), calcium acetate (ACPC_ACE), and ACP from calcium chloride (ACP_CL) and calcium nitrate (ACP_NIT).

ACP is a metastable compound. Therefore, sterilization and long-term stability are addressed in the current research. The major focus of this study was developing a simplified ACPC synthesis method and comparison of the physiochemical properties of the final products (ACP and ACPC).

## 2 Materials and Methods

Calcium citrate tetrahydrate, calcium chloride, trisodium phosphate, and sodium hydroxide were procured from Sigma Aldrich. Calcium acetate monohydrate and calcium nitrate tetrahydrate were procured from Honeywell, Fluka, and VWR chemicals BDH.

### 2.1 Synthesis of ACP and ACPC

The synthesis of ACP and ACPC were performed at an ambient temperature (around 20 to 22 °C) and pH close to 11.5. For ACP_CL, ACP_NIT, ACPC_ACE, and ACPC_CIT, calcium chloride, calcium nitrate, calcium acetate, and calcium citrate were utilized, respectively, as calcium sources. For ACP_CL, ACP_NIT, and ACPC_ACE, the concentration of the calcium source was 150 mM, whereas, for ACPC_CIT, the concentration of the calcium source was 50 mM, and for all of the reactions, the concentration of trisodium phosphate was set to 100 mM. Both calcium and phosphate salt solutions were prepared in Milli-Q water and kept on separate magnetic stirrers at 500 rpm. To adjust the pH to 11.5, a few drops of 3 M sodium hydroxide were added to the beaker containing respective calcium salts. Further, a trisodium phosphate solution was rapidly added to the stirred calcium salt solution, and the reaction was performed at a pH of 11.5. Immediately after the precipitation, the suspension was centrifuged at 3000 rpm for 5 min and washed thrice with Milli-Q water. The reaction was stopped by immersing centrifuge tubes containing the precipitated ACPs and ACPCs in liquid nitrogen for 15 min. Excess water from the frozen precipitates was removed by freeze-drying (72 h). The obtained powder was preserved in airtight containers for further characterization.

### 2.2 Characterization of ACP and ACPC

An X-ray diffractometer (PANalytical X’Pert PRO MPD) equipped with a Cu tube (Cu Kα = 1.54 Å) was used to record the diffraction patterns of the synthesized powders. Diffraction data were collected at 40 kV and 30 mA in a step mode with a step size of 0.04°, in the 2θ range from 10° to 70°. Samples were prepared by gently packing the powder on a zero-background sample holder.

Fourier-transform infrared (FTIR) spectroscopy was performed in transmission mode from the wavenumber ranging from 4000 to 400 cm^–1^ with a resolution of 4 cm^–1^ (64 scans) using a Thermo Scientific Nicolet iS50 FT-IR spectrometer.

Raman spectral acquisition was recorded using a confocal Raman microscope (Renishaw plc) equipped with a 514 nm laser. The analysis was performed using a 50x objective to focus the excitation beam and collect the backscattered signals from the samples. The spectral scan was analyzed from 350 to 2000 cm^–1^ with three-time accumulation and an exposure time of 20 s. The system was calibrated at 520 cm^–1^ against a silicon wafer and periodically checked during the experiments to ensure the accuracy of the Raman shifts.

Solid-state ^31^P NMR analysis was recorded by a JOEL, ECZR 600 MHz NMR spectrophotometer. The experiment was performed with a single 90° pulse at a mass frequency of 10 kHz. The number of scans was 338, and the relaxation delay was 5 s. Solid-state ^13^C NMR spectra was recorded by a Bruker 18.8 T, 800 MHz NMR spectrophotometer. ^13^C spectroscopy was performed with a single 90° pulse at a mass frequency of 10 kHz with 2048 scans, and the relaxation delay was 3 s.

X-ray photoelectron spectroscopy (XPS) was used to analyze the chemical composition of the samples. The spectrometer was of ThermoFisher Escalab 250xi. The pressure during spectra acquisition with the charge neutralizer switched on was 10^–7^ mbar. The calibration and linearity of the binding energy scale were confirmed by measuring the positions of Ag 3d_5/2_, Au 4f_7/2_, and Cu 2p_3_ to be at 368.21, 83.93, and 932.58 eV, respectively. The full-width at half-maximum (FWHM) of the Au 4f_7/2_ peak was better than 0.58 eV. The size of the analyzed sample was 650 mm × 100 mm.

The true density of ACP and ACPC was measured by a helium pycnometer Micro UltraPyc 1200e (Quantachrome instruments). Before measurement, calibration was performed by using a stainless steel calibration sphere. After calibration, a known amount of ACP or ACPC powder was added to the sample holder and purged with helium gas in pulse mode (30 pulses). Further, the volume was analyzed by pressurizing the sample with helium gas at a 10 psi pressure. The sample weight with the analyzed sample volume was used to calculate the true density. The analysis of each ACP and ACPC was performed in triplicate.

A nitrogen adsorption system Quadrasorb S1 (Quantachrome instruments) was used to determine the specific surface area (SSA) of the synthesized ACP and ACPC powder by the Brunauer–Emmett–Teller (BET) method. For removal of moisture, degassing of samples was performed for 24 h at room temperature before the analysis.

The morphology and particle size of synthesized ACP and ACPC were evaluated by a FEG-TEM (Tecnai G2 F30) operated at 300 kV. The sample preparation was as follows: a small amount of powder was dispersed in isopropyl alcohol and sonicated in an ultrasonic bath. Further, the samples were placed on a carbon-coated grid and dried before analysis.

### 2.3 Ion Release

Sample preparation for ion release studies was as follows: 50 mL of a 1% w/v ACP or ACPC suspension was prepared in Milli-Q water and incubated at 37 °C under constant stirring (250 rpm). Before analysis, the suspension was centrifuged at 3000 rpm for 5 min, and 750 μL of the supernatant was removed. The ACPs and ACPCs were further incubated in water at 37 °C under constant stirring until the next time point. The study was conducted for 7 days, and time points were recorded at 1, 24, 72, 120, and 168 h, respectively.

Calcium ion release was determined by a colorimetric calcium kit (Sigma Aldrich). The concentration of calcium ions was measured by the chromogenic complex between calcium ions and *o*-cresol phthalein, which is proportional to calcium ion concentration. Then, 50 μL of the supernatant was added to 96-well plates, in which 90 μL of chromogenic reagents and 60 μL of calcium assay buffer were added and gently mixed. The reaction was conducted for 5 min at room temperature in the dark, and further absorbance of the sample was recorded at 575 nm using a microplate reader (PHOmo, Anthos Mikro Systeme GmbH).

Orthophosphate ion release was evaluated by an orthophosphate calorimetric kit (Sigma Aldrich). Orthophosphate reacts with a chromogenic complex and produces a calorimetric product proportional to the orthophosphate concentration. Two hundred microliters of the supernatant was added to 96-well plates, in which 30 μL of the phosphate reagent was added and gently mixed. The reaction was conducted for 30 min at room temperature in the dark, and further absorbance was recorded at 650 nm using a microplate reader (PHOmo, Anthos Mikro Systeme GmbH).

### 2.4 In Vitro Biocompatibility Assay

An osteoblast precursor cell line derived from mouse (*Mus musculus*) calvaria (MC3T3-E1) was employed for cellular analysis after 10 passages. MC3T3-E1 cells were maintained in an α-MEM medium containing 10 vol % fetal bovine serum (Gibco Life Science) and 10 vol % penicillium–streptomycin at 37 °C in a humidified atmosphere of 95% air and 5% CO_2_. The cultures of MC3T3-E1 cells were trypsinized, counted, and 1 × 10^5^ cells/mL were inoculated into a 24-well plate followed by incubation at 37 °C in a humidified atmosphere of 95% air and 5% CO_2_ for 24 h.

Due to the metastable properties of ACP, heat sterilization was not possible. Therefore, a sterile synthesis approach was developed and utilized in this study. Solutions of 150 mM calcium acetate, calcium chloride, and calcium nitrate, 100 mM trisodium phosphate, and 3 M sodium hydroxide were sterile filtered through 0.22 μm pore size filters. Then, 50 mM calcium citrate powder and 500 mL of Milli-Q water were sterilized by autoclaving at 121 °C at 15 psi for 30 min. Under the flow cabinet, the synthesis of ACP and ACPC was performed by the procedure described in Section 2.1 with all of the sterile precursors.

For cellular analysis, suspensions were prepared by adding a 10 w/v% ACP or ACPC precipitate in an α-MEM medium and incubating at 37 °C in a humidified atmosphere of 95% air and 5% CO_2_ for 24 h. The extracts were collected by centrifugation and filtered to eliminate solid particles. The extracts were further diluted with the α-MEM medium to get the desired concentrations of 1 and 0.1 w/v%. Therefore, the total sample concentration comprises 10, 1, and 0.1 w/v% each ACP and ACPC. The extracts were then added to MC3T3-E1-containing well plates and incubated for 48 h. The α-MEM medium was added as a positive control, whereas the α-MEM medium with 6 vol % DMSO (dimethyl sulfoxide) was utilized as a negative control. Each sample was prepared in triplicate, and the same procedure was performed for all ACPs and ACPCs.

A WST-8 (CCK-8, Sigma Aldrich) kit was used to analyze the cell viability. In a colorless α-MEM medium, 1 v/v% WST was prepared. To each well, 400 μL of the 1 v/v% WST mixture was added and incubated for 3 h. The WST solution was used as a blank. Further, 100 μL of an aliquot from each 24-well plate was transferred to a 96-well plate. For spectrophotometric analysis, a 96-well plate was assigned in a microplate reader (PHOmo, Anthos Mikro Systeme GmbH), and absorbance was recorded at 450 nm. The experiments were performed in triplicate and cell viability was calculated from eq 1
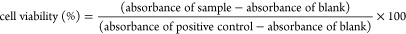
1

### 2.5 Cell Morphology

The cellular morphology of MC3T3-E1 was analyzed by hematoxylin and eosin (H&E) staining. After removing the WST solution from the 24-well plate, wells were washed with phosphate saline buffer (PBS) and fixed with 4% paraformaldehyde in PBS for 15 min. Further, it was washed with PBS and stained for 10 min with hematoxylin. Subsequently, samples were washed with tap water and then with Scott’s water, followed by washing with deionized water for 5 min. Later, samples were stained with eosin (0.1 wt/v % eosin, 5% v/v% acetic acid, 60 v/v% ethanol, and 35 v/v% ultrapure water) for 5 min. Afterward, samples were washed with 95 v/v% ethanol and 99.5 v/v% ethanol and dried at room temperature. The cell morphology was analyzed using an optical microscope (Primo Vert, Carl Zeiss).

### 2.6 Statistical Analysis

Origin 2020 (Origin Lab, Northampton, MA) was utilized to perform statistical analysis by one-way ANOVA and Bonferroni’s test. Probability (*P*) values *p* < 0.05 were considered the statistically significant differences. The results are expressed in mean ± standard deviation (S.D)

## 3 Results and Discussion

### 3.1 Characterization of ACP and ACPC

#### 3.1.1 XRD

**Figure 2:**
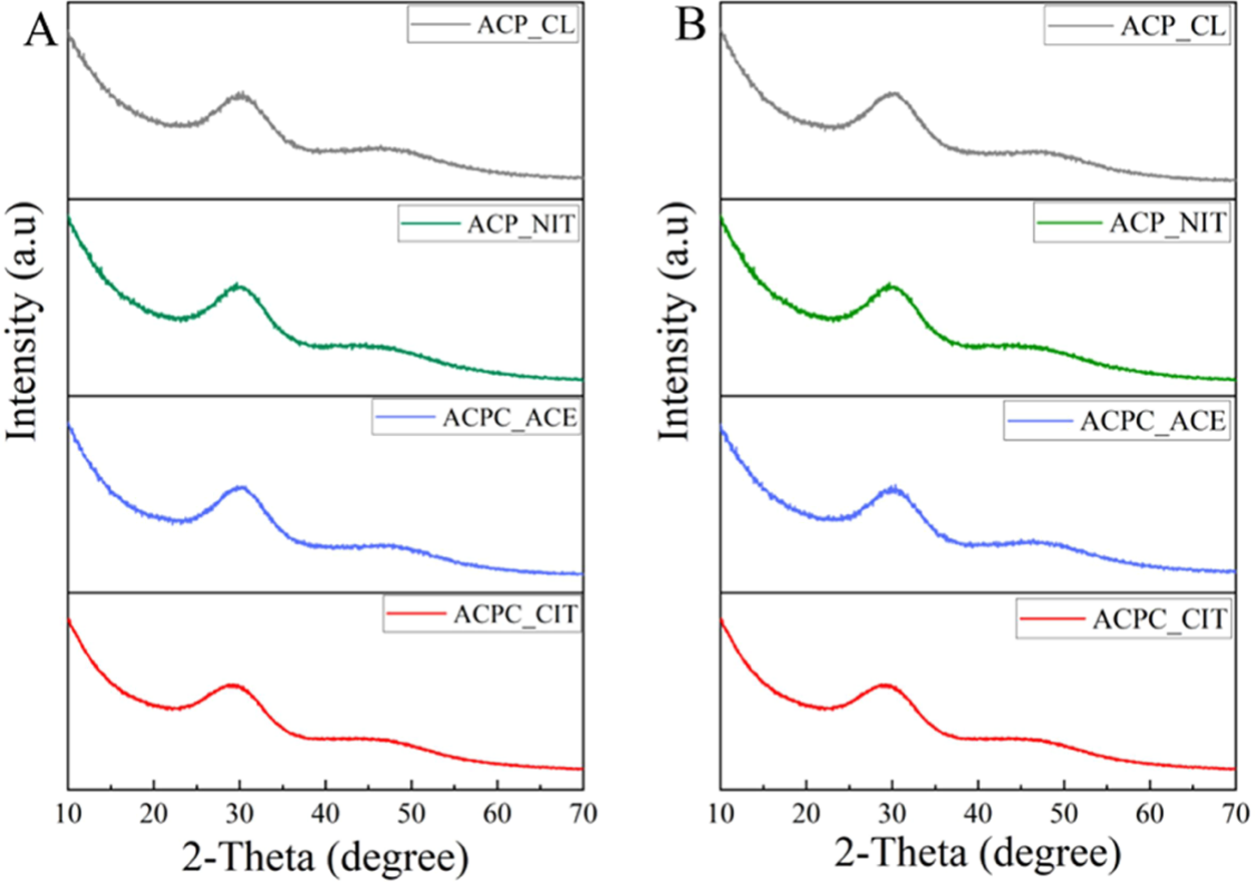
X-ray diffraction pattern of the synthesized ACPs and ACPCs. (A) Freshly prepared powder samples and (B) after 1 year of storage.

The feature that differentiates ACP from other calcium phosphates is the lack of crystalline order.^27^ As shown in Figure 2A, the XRD patterns of all of the synthesized ACPs and ACPCs were similar. As a metastable compound, ACP tends to convert into a crystalline phase; therefore, analyzing the stability of ACP is paramount. After initial characterization, the synthesized ACP and ACPC powders were stored in airtight containers, and the XRD analysis were performed after 1 year. As shown in Figure 2B, after 1 year of storage, all samples were still X-ray amorphous, thus confirming the long-term stability of all of the synthesized materials.

#### 3.1.2 Vibrational Spectroscopies

**Figure 3:**
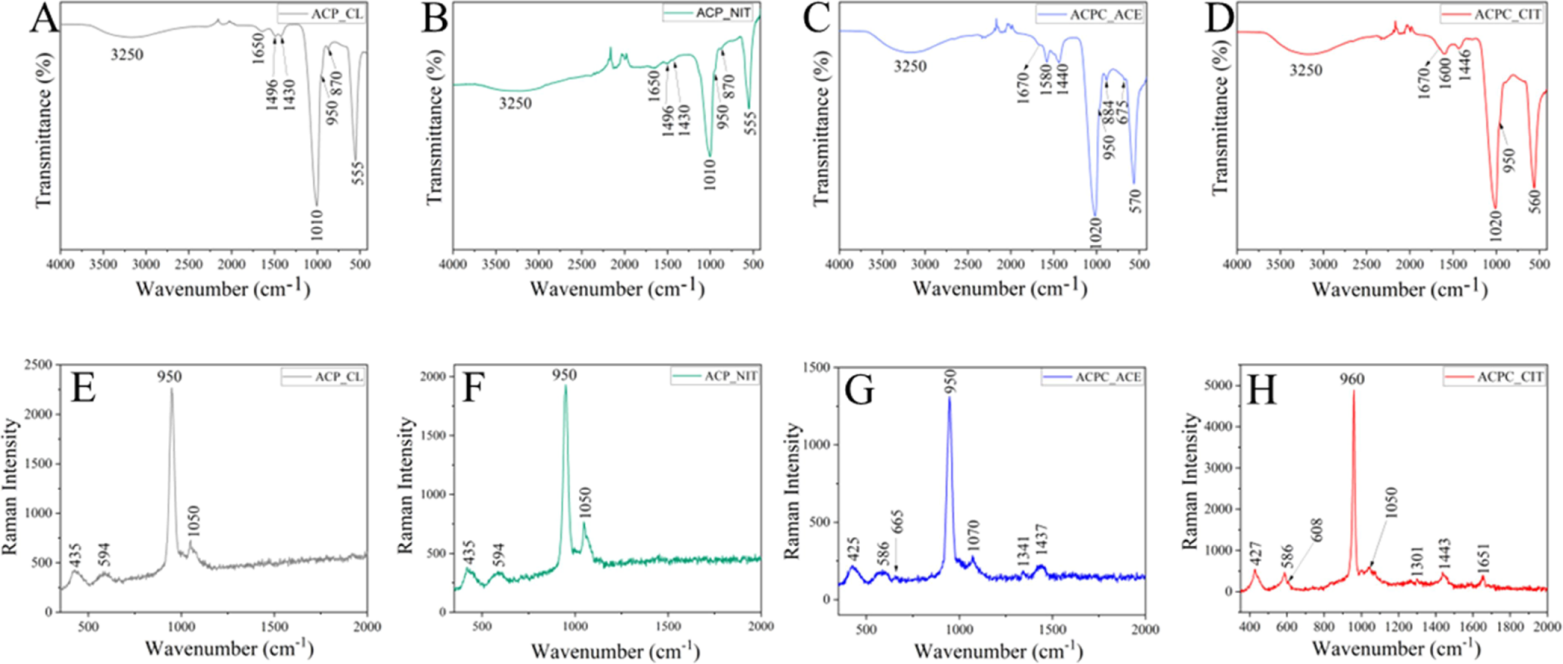
(A–D) FTIR spectra of all of the synthesized ACPs and ACPCs. (E–H) Raman spectra of all of the synthesized ACPs and ACPCs.

The characteristic IR and Raman absorption bands of all of the synthesized ACPs and ACPCs are displayed in Figure 3A–H. Water molecules have three vibration modes: asymmetric and symmetric stretching modes have very close energies, making distinguishing difficult. Therefore, a broad band of water was observed at around 3000–3700 cm^–1^. The bending mode of water was observed as a narrow band at around 1680–1640 cm^–1^.^28,29^ These bands were observed in all of the synthesized samples. In IR and Raman analyses, the PO_4_^3–^ group possesses four vibration domains: ν_1_ at around 950 cm^–1^, ν_2_ at 400–470 cm^–1^, ν_3_ at 1000–1150 cm^–1^, and ν_4_ at 500–620 cm^–1^. In IR analysis, the phosphate ν_1_, ν_2_, and ν_3_ vibrations are observed, whereas in Raman, all four PO_4_^3–^ vibrations are detected in all of the synthesized ACPs and ACPCs.

Bands associated with C–O stretches have high intensity in IR spectra, whereas those associated with C–C stretches have high intensities in Raman spectra. Moreover, the bands associated with the bending modes have moderate intensities in both IR and Raman spectra.^30^ Like phosphate, carbonate ions also possess four vibrational domains: ν_1_ at around 1050 cm^–1^, ν_2_ at 820–900 cm^–1^, ν_3_ at 1400–1550 cm^–1^, and ν_4_ at 650–750 cm^–1^. In IR spectra of ACP_CL and ACP_NIT, the stretching doublet was observed at around the 1400–1550 cm^–1^ region, corresponding to the asymmetric stretching of ν_3_ CO_3_^2–^ anions. Furthermore, the peak observed at ∼875 cm^–1^ represents the out-of-plane bending of the ν_2_ CO_3_^2–^ group. However, in the similar range at ∼875 cm^–1^, the P–OH stretching mode overlaps heavily with ν_2_ CO_3_^2–^.^31^ The peak at ∼875 cm^–1^ was absent in ACPC_CIT, which indicates the incorporation of COO^–^ in the ACP.^32^ Additionally, the bands observed at 1600 and 1432 cm^–1^ represent the COO^–^ bending and COH stretching of the carboxylic group in citrates.^30^ Likewise, in ACPC_ACE, the band revealed at 675 and 1550 cm^–1^ corresponds to COO^–^ bending and stretching, and 1440 cm^–1^ represents COH stretching of the acetate carboxylic group.^33^ The band observed at 884 cm^–1^ indicates shifting of the HPO_4_^2–^ group, which may be due to the association of the carboxylic group.^32^

In the Raman spectra of ACPC_ACE, the bands observed at 1341 and 1437 cm^–1^ indicate H–C–H deformation and COO^–^ stretching of acetate. The O–C–O bending of acetate is observed in the region of 600–680 cm^–1.^^34^ Acetate is a mono-carboxylic anion, whereas citrate is a tricarboxylic anion; therefore, the Raman spectra of ACPC_CIT were more complex. In ACPC_CIT, the sharp peak observed at 960 cm^–1^ may represent two functional groups: ν_1_ PO_4_^3–^ and/or the CH_2_ rocking vibration of citrate.^22,30^ This might be a reason for shifting of the ν_1_ PO_4_^3–^ band from 950 to 960 cm^–1^. The characteristic carboxylic band was observed at 1443 cm^–1^, and the band at 1651 cm^–1^ represents COO^–^ vibration coupled with CH_2_ bending vibration observed at 1301 cm^–1^. The out-of-plane COO^–^ modes can be assigned to the region 500–800 cm^–1^, which can be attributed to the peaks observed at 586 and 608 cm^–1^, respectively.^30^ Solid-state NMR analysis was performed to confirm the presence of acetate and citrate in ACPC_ACE and ACPC_CIT.

#### 3.1.3 NMR Analysis

**Figure 4:**
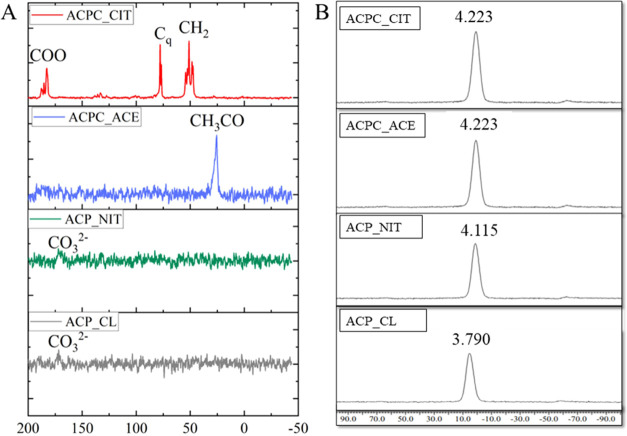
Solid-state NMR analysis of synthesized ACPs and ACPCs: (A) solid-state ^13^C NMR spectra and (B) solid-state ^31^P NMR spectra.

The ^13^C and ^31^P NMR spectra of the synthesized ACPs and ACPCs are presented in Figure 4A,B, respectively. In the ACPC_CIT samples, three carboxylate signals were observed; COO(1) corresponds to a strong signal at 182 ppm, and COO(2) and COO(3) are represented by 183 and 185 ppm, respectively. The signal at 77 ppm represents the quaternary carbon Cq and the second signal at 76 ppm indicates the association of the Ca^2+^ ion with −OH of the Cq in citrate.^35^ The methylene groups CH_2_(1) and CH_2_(2) are represented at 51 and 47 ppm, respectively. This indicates the incorporation of citrate in ACP.^15,16,18^ The signal observed at 26 ppm in ACP-ACE corresponds to the acetyl (CH_3_CO−) group of acetate.^36,37^ The ^13^C NMR analysis confirmed the retention of citrate and acetate in the ACPC_CIT and ACPC_ACE, respectively. For samples ACP_NIT and ACP_CL, a small signal was observed at 170 ppm, which corresponds to the CO_3_^2–^ group.^38^

The ^31^P spectra of ACPs and ACPCs are shown in Figure 4B. The characteristic broad Gaussian-shaped signal is between −15 and 15 ppm, centered from 2.2 to 6.5 ppm.^39−42^ This area represents the PO_4_^3–^ a resonance observed in all of the synthesized ACPs and ACPCs. The signal observed at 4.223 ppm in ACPC_CIT and ACP-ACE was identical; this can be due to the association of the COO^–^ group. On the other hand, ACP_NIT and ACP_CL show a broad peak at around 4.1 and 3.7 ppm, respectively.

#### 3.1.4 XPS

**Figure 5:**
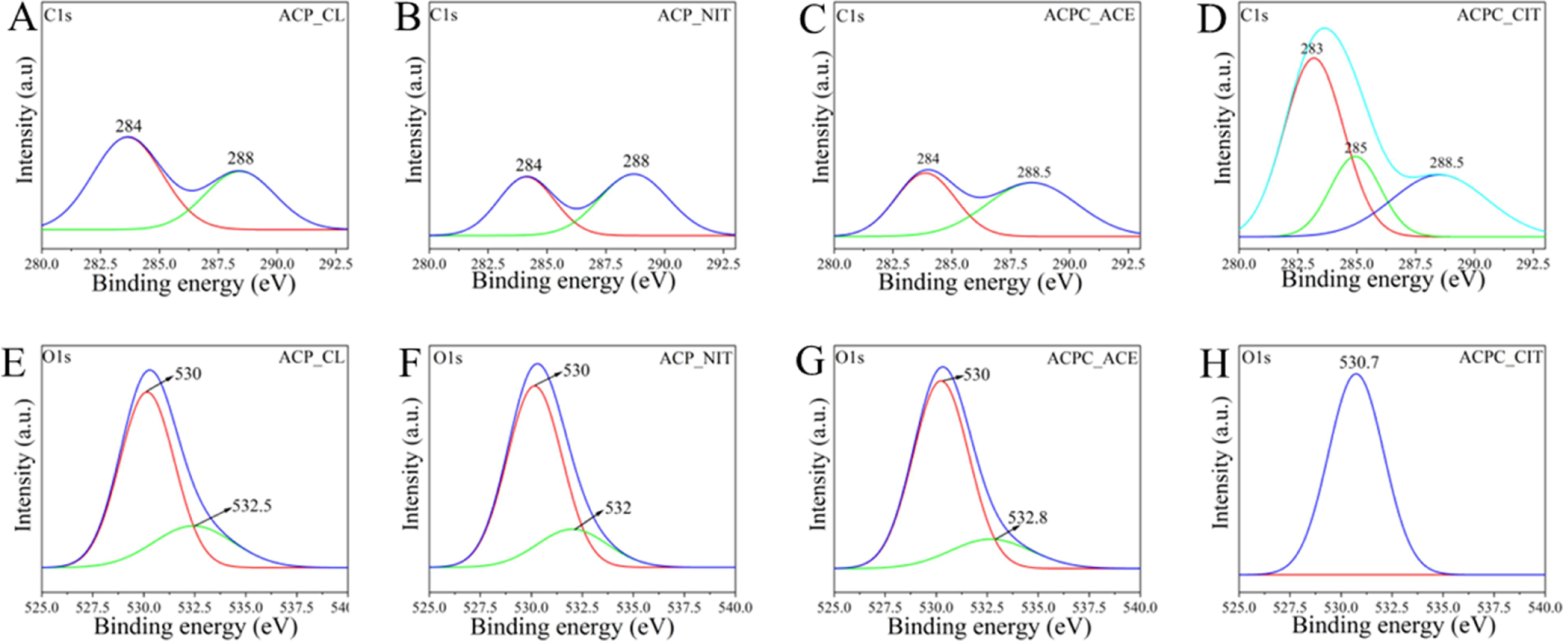
XPS of all of the synthesized ACPs and ACPCs (A–D) focuses on C1s spectra in the binding energy range of 280–292.5 eV and (E–H) focuses on O1s spectra in the 525–540 eV binding energy range. Spectral lines are represented as smoothened, normalized to their maxima, and deconvoluted.

The fingerprint region of all of the ACP and ACPC samples is presented in Figure S1 provided in supplementary data. The obtained peaks were deconvoluted using Origin 2020 software and presented in Figure 5. The sample prepared for XPS analysis was in the form of pellets. As shown in Figure 5A–B ACP_CL and ACP_NIT, peaks at around 284 eV and 288 eV denote C–C and O–C=O of carbonates.^43^ On the contrary, the ACPC_CIT and ACPC_ACE show the presence of COO^–^ groups. Therefore, in Figure 5C, ACPC_ACE shows the absorption band at 284 eV and 288.5 eV represents the acetyl group.^44^ Typically citrate-containing compounds show three peaks at around 283, 285, and 288.5 eV. The peak at 283 eV represents the C–C and (CH_2_)*_n_* bonds, the peak at around 285 eV indicates C=O, and the third peak at around 288.5 eV accounts for COO.^45,46^ As shown in Figure 5D, these three signature carboxylate peaks were observed in ACPC_CIT samples.

The O1s spectra shown in Figure 5E–H were used to detect the presence of the HPO_4_^2–^ group. The O1s spectrum was dissymmetrical in the presence of HPO_4_^2–^ and symmetrical in the case of PO_4_^3–^. In Figure 5H, ACPC_CIT shows a symmetrical peak representing PO_4_^3–^. On the other hand, in Figure 5E–G, dissymmetry was observed in ACPC_CL, ACP_NIT, and ACP_ACE samples, confirming the presence of both PO_4_^3–^ and HPO_4_^2–^ groups.^47^

#### 3.1.5 Density and Brunauer–Emmett–Teller (BET) Analysis

The data shown in Table 1 reveals the specific surface area and density of synthesized ACPs and ACPCs. The specific surface area of the ACP_NIT and ACP_CL was the same, but a difference was observed in ACPC_CIT and ACPC_ACE. This might be due to the association of the COO^–^ group. On the contrary, there were differences in the densities of the synthesized ACPs and ACPCs. This shows that the nature of COO^–^ ions affects the properties of ACP.

**Table 1:** Density, BET, and Average Particle Size of the Synthesized ACPs and ACPCs from Different Calcium Sources

sample	density (g/cm^3^)	BET (m^2^/g)
ACP_CL	2.62	105
ACP_NIT	2.58	105
ACPC_ACE	2.47	118
ACPC_CIT	2.57	62

#### 3.1.6 FEG_TEM Analysis

**Figure 6:**
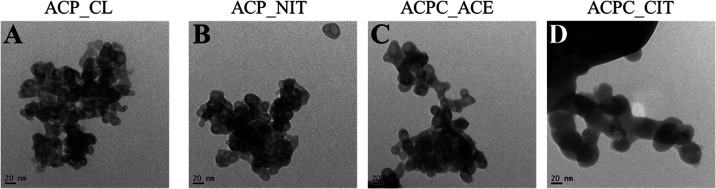
Morphological evaluation of ACP and ACPC synthesized from different calcium precursors. The effect of different calcium precursors was observed on the particle size of ACP. The samples are represented as (A) ACP_CL, (B) ACP_NIT, (C) ACPC_ACE, and (D) ACPC_CIT. Scale bar: 20 nm.

The morphology of the synthesized ACPs and ACPCs was evaluated by FEG-TEM, as shown in Figure 6A–D. The sample ACPC_ACE, ACP_CL, and ACP_NIT showed porous spherical particles of a size less than 20 nm. On the contrary, ACPC_CIT reveals solid particles of size ∼40 nm. Previous studies have reported that ACP nanoparticles had maximum stability with a diameter of 30–50 nm.^48^ Naturally, ACP is synthesized and stabilized in mitochondria, nucleates in the 40 nm collagen gap zone, and converts to apatite.^49^ The elongated plate-like geometry of apatite has a length between 30 and 50 nm and a width between 15 and 30 nm while maintaining a thickness of 2–10 nm.^50^ Therefore, the ∼40 nm particle size of ACPC_CIT is in the size range to fit in the gap zone of collagen. Moreover, the association of citrate provides sites (−CH_2_) for binding with nonpolar amino acids such as alanine and proline in the collagen matrix. ACPs are highly sensitive to the electron beam and crystallize rapidly under high-energy electron irradiation exposure.^49^ The crystallization of both ACP and ACPC particles under a high-energy electron beam was observed and can be seen in Figure S2 in the supplementary data.

#### 3.1.7 Ion Release

**Figure 7:**
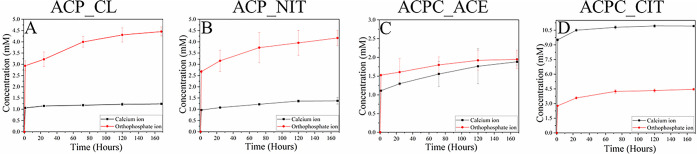
Kinetic release of phosphate and calcium ions in (A) ACP_CL, (B) ACP_NIT, (C) ACPC_ACE, and (D) ACPC_CIT was performed for 168 h (7 days).

The kinetics of ion release from the ACPs and ACPCs is shown in Figure 7A–D. The ion release was studied for 168 h (7 days) to analyze Ca^2+^ and phosphate ion release. A burst release was initially observed within the first hour, gradually reducing over time.^51,52^ The highest ion release was observed in ACPC_CIT, followed by ACP_NIT and ACP_CL, and the least was in ACPC_ACE. Moreover, the release of Ca^2+^ ions was more than the phosphate ions in ACPC_CIT, whereas the opposite scenario was observed in all other ACPs. In Figure 7A,B, in ACP_NIT and ACP_CL, the phosphate ion release was between 3 and 4 mM, whereas the calcium release was between 1 and 2 mM. Comparatively, the Ca^2+^ and phosphate ions released in ACPC_ACE ranged between 1 and 1.5 mM. Acetate possesses fewer COO^–^ groups than citrate, so the ion release might differ from ACPC_CIT. From these studies, it can be determined that ACP and ACPC possess different ion release profiles. Additionally, different COO– groups have a considerable effect on the ion release profiles of ACPC.

### 3.2 Cellular Analysis

#### 3.2.1 In Vitro Cytotoxicity

**Figure 8:**
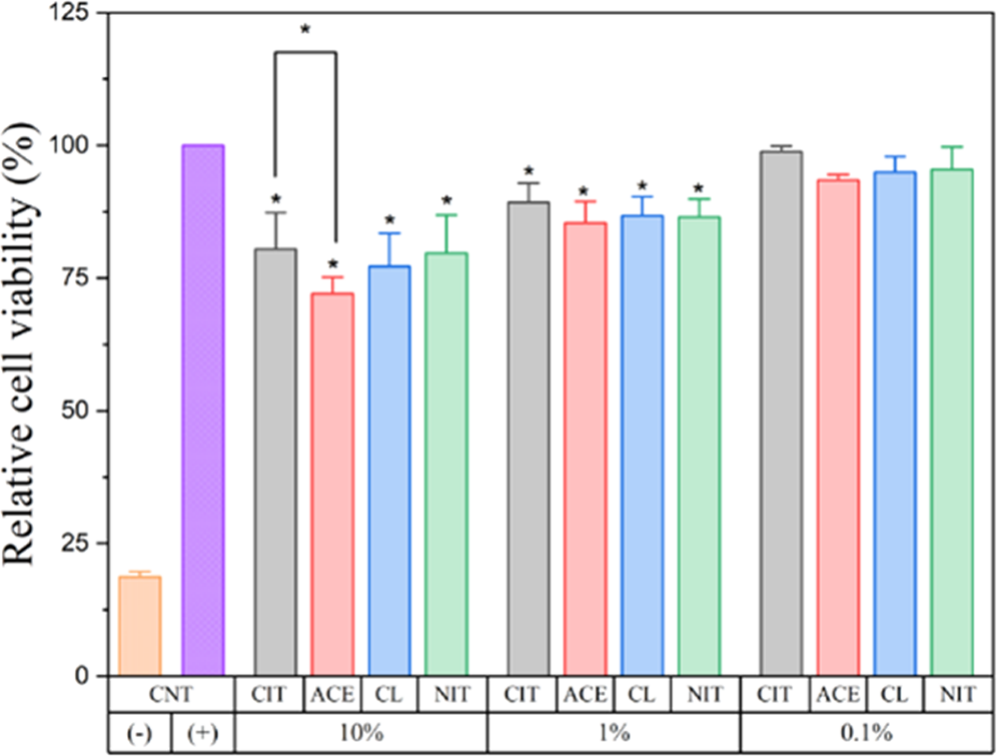
Relative viabilities of MC-3T3E1 cells cultured with the extract of different ACPs and ACPCs in 10%, 1%, and 0.1% w/v cell culture media dilutions (*n* = 12, CNT = control, samples in triplicate, *p < 0.05). CIT, ACE, CL, and NIT represent the ACP samples ACPC_CIT, ACPC_ACE, ACP_CL, and ACP_NIT, respectively.

The cell viability of MC-3T3E1 cells in the presence of extracts of ACPs and ACPCs is shown in Figure 8. The absorbance recorded from the positive control cells cultured in the only medium was normalized as 100%. The cells cultured with a 10 w/v% extract of ACPC_ACE showed the lowest cell viability, whereas the highest cell viability was observed in a 0.1 w/v% extract of ACPC_CIT. In the group of 10 w/v%, ACPC_CIT possesses the highest cell viability, followed by ACP_CL, ACP_NIT, and ACPC_ACE. A similar trend was observed in 1 and 0.1 w/v% ACP extracts. The extract of 10 w/v% ACP_CL, ACP_NIT, and ACPC_ACE maintained a cell viability of ∼80%, whereas for ACPC_ACE, it was close to ∼72%. However, extracts of respective ACP and ACPC with 1 and 0.1 w/v% extracts show cell viabilities of ∼90 and ∼95%, respectively. It can be concluded that ACPC_CIT has more cell viability in all of the respective groups. Overall, after 48 h of cell culture, the cell viability increased gradually with a higher dilution of ACP and ACPC. Since all of the ACP and ACPC samples show cell viability of more than ∼70%, it can be inferred that all of the samples were cytocompatible. In vitro analysis indicates that ACPC_CIT possesses maximum cell viability compared to other ACP and ACPC samples, indicating that the association of citrates enhanced the cell viability of ACP.

#### 3.2.2 Cell Morphology

**Figure 9:**
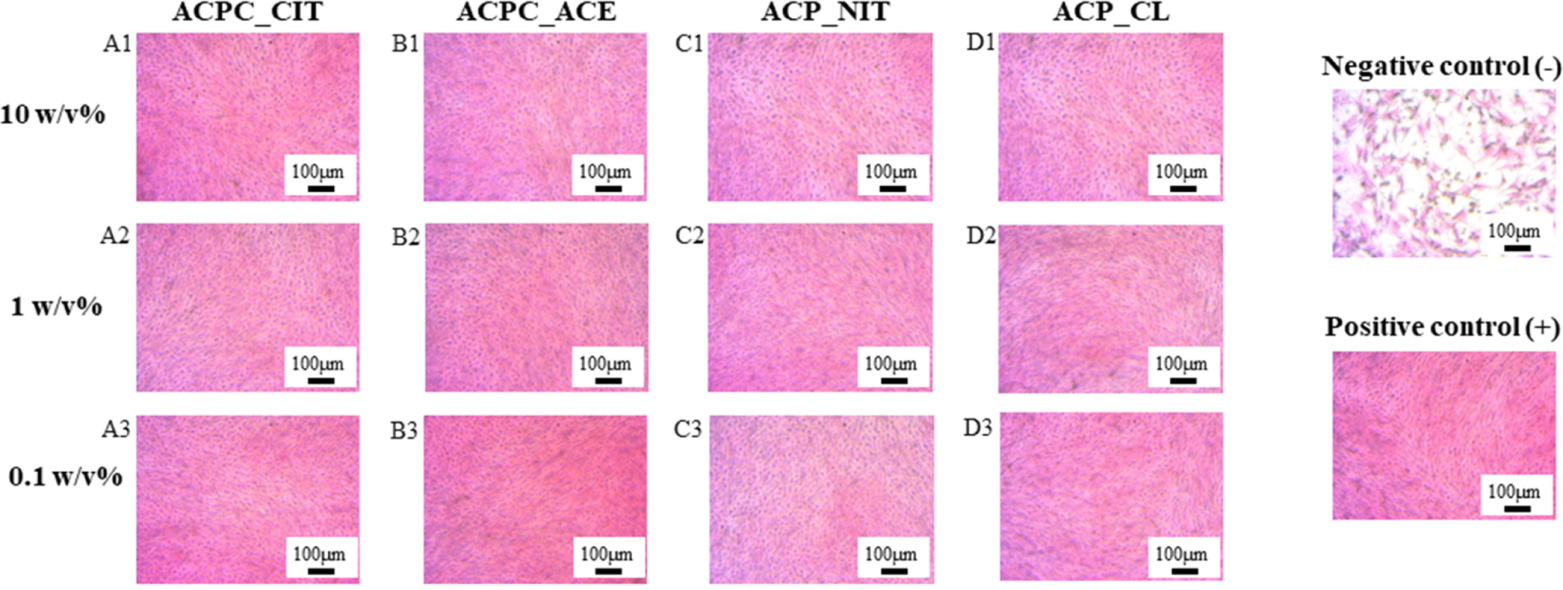
Optical microscopy was performed on H&E-stained MC-3T3E1 cells cultured with the extract of different ACP and ACPC. Cells treated with 10 wt % extracts of ACPC_CIT, ACPC_ACE, ACP_NIT, and ACP_CL are displayed from A1 to D1, respectively. Panels from A2 to D2 indicate cells treated with 1 wt % extracts of ACPC_CIT, ACPC_ACE, ACP_NIT, and ACP_CL, respectively. Panels from A3 to D3 denote the cells treated with 0.1 wt % extracts of ACPC_CIT, ACPC_ACE, ACP_NIT, and ACP_CL, respectively.

Optical microscopy images of H&E-stained MC-3T3E1 cells cultured with 0.1, 1, and 10% w/v extracts of synthesized ACPs and ACPCs are shown in Figure 9. The shape and size of the cells were not affected in the presence of ACP and ACPC extracts; thus, the cytocompatibility was not affected. Moreover, the results were correlated with the cell viability measured by the WST-8 assay, which confirms the biocompatibility of all of the synthesized samples. The image of H&E staining at lower magnification is displayed in Figure S3 provided in supplementary data.

## 4 Discussion

Citrate is tricarboxylic acid naturally associated with bone.^15,16,57−60^ Moreover, citrate is also known to stabilize ACP.^61,62^ Therefore, citrate is a natural candidate in the development of ACPC. From the biomimetic perspective, the particle size of ACP is a crucial parameter as it needs to ft in the 40 nm gap zone of collagen. Studies in zebrafish, developing mouse calvaria, and chicken long bone have shown the presence of ACP in the size range of 50–80 nm.^49^ However, synthesizing ACPs in this range is challenging.

In the literature, synthetic ACPs show size variations ranging from several nanometers to micrometers. The synthetic reactions of ACPs are highly sensitive to pH, temperature, and precursor concentration, affecting particle size. A brief literature review in Table S1 in supplementary data shows variations in particle sizes and synthesis pH of ACP. Moreover, most previous studies utilized calcium chloride and calcium nitrate as starting materials; however, different particle sizes were obtained. In wet synthesis, calcium chloride and calcium nitrate undergo complete dissociation. On the contrary, calcium acetate and calcium citrate undergo partial dissociation.^63^ Therefore, the mechanism of ACP formation is simple in the case of calcium chloride and calcium nitrate, whereas complex in the case of calcium acetate and calcium citrate. This partial dissociation of calcium acetate and calcium citrate may be responsible for the complex ternary formation of ACPC with functional groups of citrate and acetate.^64,65^ The association of the carboxylic group in ACP affects the physiochemical properties such as the surface area, particle size, and ion release kinetics. Moreover, citrate-associated ACP possesses biomimetic particle size.

Comparative analysis of ACP and ACPC provided new insights. First, the presence of both CO_3_^2–^ and HPO_4_^2–^ in ACP_CL and ACP_NIT. Second, the incorporation of COO^–^ ions in ACP was concluded from missing and shifting of the HPO_4_^2–^ peak at 875 cm^–1^ in IR spectra of ACPC_CIT and ACPC_ACE, respectively. Further, incorporating COO^–^ ions restricted the incorporation of the CO_3_^2–^ group, which was discovered from ACPC_CIT and ACPC_ACE, respectively. Lastly, acetate is a mono-carboxylic ion, whereas citrate is a tricarboxylic ion that greatly affects the physiochemical properties of ACP.

There are four interpretations for the interaction of citrate with calcium phosphate. The first most accepted interpretation is the interaction of Ca^2+^ with the COO^–^ group of citrates.^66^ The second interpretation is the interaction of OH^–^ of citrate with the phosphate ions.^67^ The third possibility occurs by substituting the phosphate group with a citrate anion.^68^ At the synthesis pH above 11, citrates are present in the form of Hcit^3–^ ions, which can potentially substitute PO_4_^3–^ ions as they share the same charge. The fourth prediction is the interaction of carboxylic groups with phosphate ions.^69^ Focusing on the fourth prediction, carboxylates and phosphate are Lewis acids with a common preferential stereochemistry.^70^ The syn or antistereochemistry is observed in carboxylate–Lewis acid interactions. The syn interaction is preferred by carboxylate with a covalently bonded proton, metal ion, or hydrogen bond donor. The preferential reaction occurs in the plane of carboxylate to complex sp^2^ oxygen lone pair.^71^ Phosphates can react with other phosphate groups by forming P–O–H–O–P, whereas carboxylate can react with other carboxylates by C–O–H–O–C. Similar phosphate and carbonates can react with each other via P–O–H–O–C bonding, though the bond length of P–O–H–O–C is greater than that of P–O–H–O–P, indicating a slightly weaker bond. The polar nature of C–O is less than the P–O linkage, which signifies that C–O–H–O–C is even weaker.^72^ Therefore, the carboxylic groups of citrates can interact with phosphate via P–O–H–O–C bonding.

On the contrary, the mechanism of ACPC_ACE formation differs from that of ACPC_CIT. One of the main reasons is the incompatible negative charge of −1 and the lack of a secondary carboxylic group in acetate.^73^ Therefore, the association of the acetyl group with ACP can be in two different ways, either by adsorption on the ACP surface^74^ or by the formation of calcium acetyl phosphate.^75^ However, more advanced analysis is required to understand the exact interaction of ACP with carboxylates.

## 5 Conclusions

A simplified synthesis approach was developed for the synthesis of ACP and ACPC. Characterization of the synthesized materials by vibration spectroscopy, NMR, FTIR, and XPS analyses confirmed the formation of ACP and ACPC. The particle size, specific surface area, and ion release profile of the synthesized ACPs and ACPCs depend on the calcium source used. A sterile synthesis method was developed and utilized for ACP and ACPC analysis in vitro. In vitro results confirmed the biocompatibility of all of the synthesized ACPs and ACPCs. ACPC_ACE shows relatively less cell viability at a 10 w/v% concentration, whereas ACPC_CIT shows higher cell viability than other of the synthesized ACP/ACPC. The association of ACP with acetate and citrate is a complex process, and more advanced analysis is required to depict the exact interaction. All of the analyses in this study indicated the difference in the physiochemical properties of ACP and ACPC. Additionally, the physiochemical properties of ACP are affected by the incorporated carboxylic group.

## Supporting Information Available

XPS survey spectra and TEM images of ACPC_CIT, ACPC_ACE, ACP_CL, and ACP_NIT; optical microscopy of H&E-stained MC-3T3E1 cells cultured with the extract of different ACPs; and table of literature survey on the synthesis of ACP by precipitation (PDF)
