# Supplementary material for: Amorphous Calcium Phosphate and Amorphous Calcium Phosphate Carboxylate: Synthesis and Characterization
Source: ACS Omega. 2023 Jul 17;8(30):26782–92. doi: 10.1021/acsomega.3c00796 (PMC10399191; doi:10.1021/acsomega.3c00796)
Supplement: Supplementary file 1 — ao3c00796_si_001.pdf [file ao3c00796_si_001.pdf]

Amorphous calcium phosphate and amorphous calcium phosphate carboxylate: synthesis and characterization

Abhishek Indurkar[1,2], Rajan Choudhary[1,2], Kristaps Rubenis[1,2], Mansingraj Nimbalkar[3], Anatolijs Sarakovskis[4], Aldo R. Boccaccini[5,] and Janis Locs[1,2*]

[1] Rudolfs Cimdins Riga Biomaterials Innovations and Development Centre of RTU, Institute of General Chemical Engineering, Faculty of Materials Science and Applied Chemistry, Riga Technical University, Pulka Street 3, LV-1007 Riga, Latvia

[2] Baltic Biomaterials Centre of Excellence, Headquarters at Riga Technical University, Kipsalas Street 6A, LV-1048 Riga, Latvia

[3] Department of Botany, Shivaji University, Kolhapur – 416 004 (MS), India

[4] Institute of Solid State Physics, University of Latvia, 8 Kengaraga Str., LV-1063 Riga, Latvia

[5] Institute of Biomaterials, Department of Material Science and Engineering, University of Erlangen-Nuremberg, 91085, Erlangen, Germany.

*Corresponding author - Janis.Locs@rtu.lv

Content

Figure S1. XPS survey spectra of ACPC_CIT, ACPC_ACE, ACP_CL and ACP_NIT

Figure S2. The crystallization of ACP under a high electron beam. The sample represented as A) ACP_CL, B) ACP_NIT, C) ACPC_ACE and D) ACPC_CIT.

Figure S3. Optical microscopy of H&E-stained MC-3T3E1 cells cultured with extract of different ACPs. Cells treated with 10 wt% extracts of ACPC_CIT, ACPC_ACE, ACP_NIT, and ACP_CL are displayed from A1 to D1 respectively. Figures from A2 to D2 indicate cells treated with 1 wt% extracts of ACPC_CIT, ACPC_ACE, ACP_NIT, and ACP_CL are displayed respectively. Finally, the cells treated with 0.1 wt% extracts of ACPC_CIT, ACPC_ACE, ACP_NIT, and ACP_CL are displayed from A3 to D3 respectively.

Table S1 - Literature survey on the synthesis of ACP by precipitation.

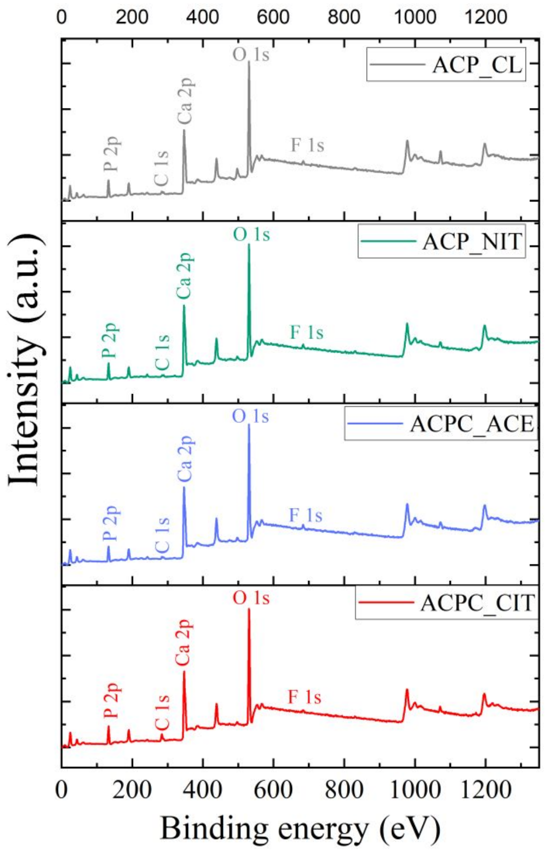

Figure S1. XPS survey spectra of ACPC_CIT, ACPC_ACE, ACP_CL and ACP_NIT

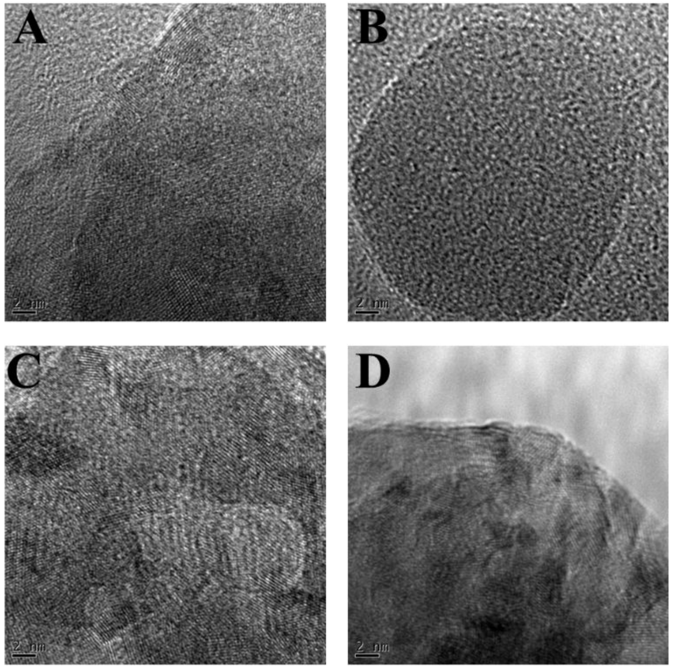

Figure S2. The crystallization of ACP under a high electron beam. The sample represented as A) ACP_CL, B) ACP_NIT, C) ACPC_ACE and D) ACPC_CIT.

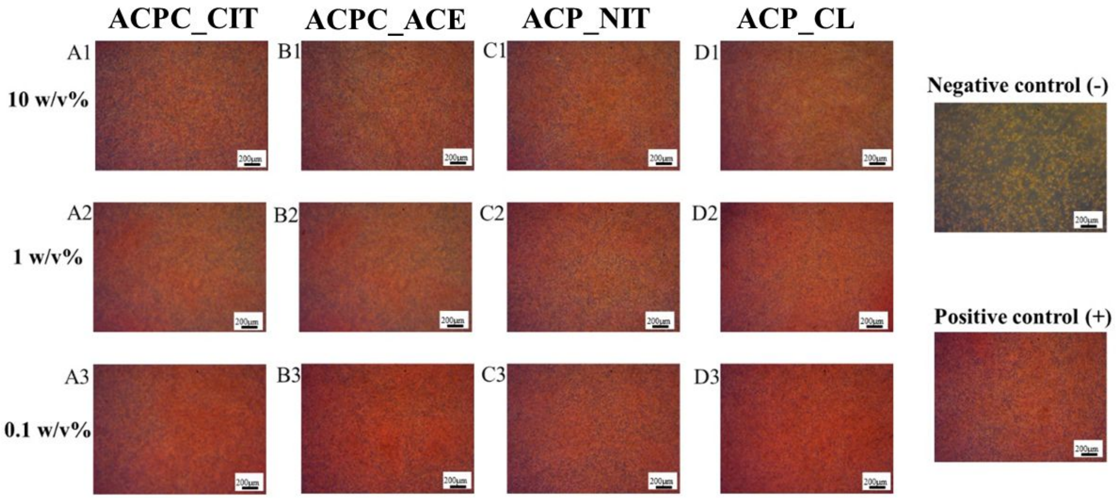

Figure S3. **Optical microscopy of H&E-stained MC-3T3E1 cells cultured with extract of different ACPs. Cells treated with 10 wt% extracts of ACPC_CIT, ACPC_ACE, ACP_NIT, and ACP_CL are displayed from A1 to D1 respectively. Figures from A2 to D2 indicate cells treated with 1 wt% extracts of ACPC_CIT, ACPC_ACE, ACP_NIT, and ACP_CL are displayed respectively. Finally, the cells treated with 0.1 wt% extracts of ACPC_CIT, ACPC_ACE, ACP_NIT, and ACP_CL are displayed from A3 to D3 respectively.**

Table S1 - Literature survey on the synthesis of ACP by precipitation.

Sr no.	Year	Calcium source	Phosphate source	Additive to achieving the desired pH	pH	Post treatments	Particle size	Cellular studies	Product

1.	2012	a. Calcium metal<br>b. $Ca(NO_3)_2.4H_2O$<br>c. $CaCl_2.2H_2O$<br>d. $Ca(OH)_2$<br>e. $Ca(CH_3COO)_2$	a. Na_2HPO_4<br>b. $(NH_4)_2HPO_4$		9.2 - 11.3	N.A.	< 70nm	N.A.	ACP [1]
2.	2017	$Ca(CH_3COO)_2$	H_3PO_4	$NH_3.H_2O$	9	Filtration and<br>Air drying	The size reported in µm of heat-treated samples	N.A.	ACP [2]
3.	2012	$CaCl_2.2H_2O$	KH_2PO_4	-	6.76	Centrifuge and Oven-dried at 37°C	Scale bar in TEM reveals size in nm. But the exact size is not indicated.	7F2 Mouse osteoblasts	ACP [3]
4.	2019	Hydroxyapatite suspension in HCl		NH_4OH	8 - 11	Centrifuge and washed with distilled water. Freeze drying or oven drying at 80°C	14 - 18 nm	N.A.	ACP [4]
5.	2018	$CaCl_2.2H_2O$	Na_2HPO_4	HCl	7.4	N.A.	56 - 85 nm	N.A.	ACP [5]
6.	2014	$Ca(NO_3)_2.4H_2O$	$(NH_4)_2HPO_4$	Ammonia	10.5 - 12	Filtration and drying at room temperature	Scale bar in TEM reveals size in nm.	N.A.	ACP [6]

							But the exact size is not indicated.		
7.	2019	Hydroxyapatite suspension in HCl		NH_4OH	8 - 11	Centrifuge and washed with distilled water. Oven drying at 80°C	11 - 30 nm	N.A.	ACP [7]
8.	2014	$CaCl_2.2H_2O$	Na_2HPO_4	-	7.4	Aging at 55°C, 65°C, and 70°C for 15 mins. Further centrifugation or filtration and drying at room temperature.	245 ± 20 nm	N.A.	ACP [8]
9.	2010	$CaCl_2.2H_2O$	$Na_3PO_4.H_2O$	NaOH	N.A.	Centrifuge and washed with distilled water. Oven drying at 60°C	10 – 50 nm	N.A.	Mg Substituted ACP [9]
10.	2017	$Ca(NO_3)_2.4H_2O$	$(NH_4)_2HPO_4$	Tris buffer	9.5	Filtration and freeze drying	260 – 300 nm	G-292 Human osteosarcoma cell line	Mg-doped ACP [10]
11.	2015	$Ca(NO_3)_2.4H_2O$	$(NH_4)_2HPO_4$	Ammonia	9.6 – 9.8	Centrifuge and washed with distilled water. Freeze drying or Oven drying at 80°C or drying at room temperature.	Scale bar in TEM reveals size in nm. But the exact size is not indicated.	MC-3T3E1 preosteoblasts	Amorphous calcium magnesium phosphate [11]

12.	2020	$CaCl_2$	$(NH_4)_2HPO_4$	NaOH	10	Filtration, washing, and drying at 60°C	< 500nm	1. SaOS-2 human osteosarcoma cell line<br>2. HUVEC human umbilical vein endothelial cells	Polyphosphate stabilized ACP [12]
13.	2016	$CaCl_2$	$Na_2HPO_4.12H_2O$	$NaBH_4$	Information not given	Hydrothermal synthesis, then the precipitate was filtered, washed and oven dried at 60°C	The doped ACP shell size was 14 nm	-	CdTe quantum dots of zerovalent iron/ACP composite [13]
14.	2016	$CaCl_2$ or $Ca(NO_3)_2.4H_2O$	$(NH_4)_2HPO_4$ or K_2HPO_4	CH_4N_2O	Information not given	Filtration washing and freeze drying	30nm	-	ACP [14]
15.	2010	$CaCl_2$	K_2HPO_4	KOH	11.5	Filtration washing and freeze drying	-	-	ACP [15]
16.	2021	$CaCl_2.2H_2O$	Na_2HPO_4	NaOH	7.8 - 11	-	-	-	ACP [16]
17.	2016	$CaCl_2$	K_2HPO_4	KOH	8	Washing and freeze drying	100nm	-	ACP [17]
18.	2021	$CaCl_2$	Na_2HPO_4	HCl	7.4	Dried in desiccator	99.5 ± 29.4nm	-	ACP [18]
19.	2022	$Ca(NO_3)_2.4H_2O$	$(NH_4)_2HPO_4$	NH_4OH	10	Filtered and washed with distilled water. Oven drying at 80°C or drying at room temperature.	Scale bar in SEM reveals size in µm. But the exact size was not indicated.	-	Zn constating ACP [19]

20.	2014	$Ca(NO_3)_2.4H_2O$	$Na_2HPO_4.12H_2O$	-	-	Centrifuge, Freeze drying, and ball milling	Scale bar in SEM reveals size in µm. But the exact size is not indicated.	-	Silver substituted ACP [20]
21.	2021	$CaCl_2.2H_2O$	Na_2HPO_4	HCl	7.4	Filtration, washing, and drying in a stream of nitrogen	64.5 ± 9.9nm to 143.5 ± 24.8 nm	-	Effect of amino acids on ACP [21]
22.	2010	$Ca(NO_3)_2.4H_2O$	$(NH_4)_2HPO_4$	NH_4OH	12	Frozen and refrozen to obtain precipitate followed by freezing and freeze drying	-	-	ACP [22]
23.	2006	$Ca(NO_3)_2.4H_2O$	H_3PO_4	-	-	-	-	Animal studies	Electro-sprayed calcium phosphate coatings [23]
24.	2014	$CaCl_2.2H_2O$	$HPO_4.3H_2O$	Tris hydroxymethyl aminomethane and HCl	7.4	Rinsed with distilled water and air dried	Particle size not given	-	Calcium phosphate on bacterial cellulose nanofibers [24]
25.	2016	$Ca(NO_3)_2.4H_2O$	Na_3PO_3S	Ammonia	10.5	Centrifuged, washed, and freeze-dried	60 – 100nm	-	Sulfur containing ACP [25]
26.	2000	$Ca(NO_3)_2$	$(NH_4)_2HPO_4$	NH_4OH	10	Washed with acetone	-	-	Effect of nickel on ACP [26]
27	2021	$CaCl_2$	H_3PO_4	-	6 to 8	-	60 and more than 500 nm	-	ACP synthesized by Epoxide Route [27]

28	2022	$CaCl_2$	NaH_2PO_4 Na_2HPO_4	NaOH	7.40	Filtered, frozen in liquid nitrogen, and freeze-dried	40 to 80 nm	-	Magnesium-doped ACP [28]
29	2021	$CaCl_2$	$(NH_4)_2HPO_4$	NaOH	8	Centrifuged, and freeze-dried	-	1. A549 cells<br>2. MG63 cells	Composite:<br>Hyaluronic acid fluorescent carbon/amorphous calcium phosphate [29]
30	2019	$CaCl_2$	H_3PO_4	-	8	Filtration, washing, and drying in a vacuum oven at 50 °C	20 to 100 nm	Mouse Bone Marrow Stem Cells	ACP synthesis [30]
31	2021	$CaCl_2$	H_3PO_4	-	8	Filtration, washing, and drying in a vacuum oven at 50°C	-	1. Mouse Bone Marrow Stem Cells<br>2. New Zealand white Rabbit	Composite:<br>ACP doped with citrate/poly-amino acid [31]
32	2020	$CaCl_2.2H_2O$	Potassium Phosphate dibasic	KOH	12	Centrifuged, washed, and stored at 4°C	Below 10 nm	-	Europium doped ACP [32]
33	2013	Biomineralization medium			7.45	Centrifugation or filtration washed and dried at room temperature	20 nm	-	ACP [33]
34	1998	$Ca(NO_3)_2.4H_2O$	$(NH_4)_2HPO_4$	Ammonia	9.8	Filtered, washed, and freeze-dried	-	-	Synthesis in alcohol medium [34]
35	2020	$Ca(CH_3COO)_2.H_2O$	H_3PO_4	-	-	Spray drying	100 nm	-	ACP [35]

36	1999	$CaCl_2$	KH_2PO_4, K_2HPO_4	Tris(hydroxymethyl)aminomethane	6.8	-	-	-	ACP [36]
37	2005	$Ca(NO_3)_2.4H_2O$	$(NH_4)H_2PO_4$ $(NH_4)_2HPO_4$	Ammonia	10	Filtered, washed, and air-dried	-	-	ACP [37]
38	2009	$CaCl_2.2H_2O$	Na_2HPO_4	NaOH	-	Centrifuged and washed	-	-	ACP [38]
39	2017	$CaCl_2$	K_2HPO_4	-	-	Filtered, washed, and freeze-dried	29-98 nm	-	ACP [39]
40	2008	$Ca_5(PO_4)_3OH$	-	NaOH	6.5	Centrifuged and washed	30-50 nm	-	ACP-chitosan [40]
41	2019	$Ca(NO_3)_2.4H_2O$	$(NH_4)_2HPO_4$	Ammonia	10	Filtered, washed, and dried at 50°C	-	-	Mn-ACP [41]
42	2013	$Ca(NO_3)_2.4H_2O$	$(NH_4)_2HPO_4$	NH_4OH	10	Centrifuged and dried at 70°C	-	-	ACP [42]
43	2011	$CaCl_2$	H_3PO_4	pH not adjusted	-	-	-	-	ACP [43]
44	2014	$Ca(NO_3)_2.4H_2O$	$(NH_4)_2HPO_4$	-	-	washed and freeze-dried	40 nm	-	ACP [44]
45	2004	$CaCl_2$	$(NH_4)H_2PO_4$	NaOH	7.1	washed and dried	-	-	ACP [45]
46	2018	$Ca(NO_3)_2$	$(NH_4)_2HPO_4$	Ammonia	10	washed and dried at 25 °Cs	34.4 nm	-	Polyacrylic acid nano Hap [46]
47	1999	$Ca(NO_3)_2$	diammonium phosphate	NH_4OH	8.5 to 9	Filter washed and dried	submicron	-	ACP to HaP [47]

References

[1] Tas AC. Calcium metal to synthesize amorphous or cryptocrystalline calcium phosphates ☆ 2012. https://doi.org/10.1016/j.msec.2012.01.024.

[2] Safronova T V., Mukhin EA, Putlyaev VI, Knotko A V., Evdokimov P V., Shatalova TB, et al. Amorphous calcium phosphate powder synthesized from calcium acetate and polyphosphoric acid for bioceramics application. Ceram Int 2017;43:1310–7. https://doi.org/10.1016/J.CERAMINT.2016.10.085.

[3] Zhou H, Bhaduri S. Novel microwave synthesis of amorphous calcium phosphate nanospheres. J Biomed Mater Res Part B Appl Biomater 2012;100B:1142–50. https://doi.org/10.1002/JBM.B.32681.

[4] Vecstaudza J, Gasik M, Locs J. Amorphous calcium phosphate materials: Formation, structure and thermal behaviour. J Eur Ceram Soc 2019;39:1642–9. https://doi.org/10.1016/J.JEURCERAMSOC.2018.11.003.

[5] Čadež V, Erceg I, Selmani A, Jurašin DD, Šegota S, Lyons DM, et al. Amorphous Calcium Phosphate Formation and Aggregation Process Revealed by Light Scattering Techniques. Cryst 2018, Vol 8, Page 254 2018;8:254. https://doi.org/10.3390/CRYST8060254.

[6] He K, Xiao GY, Xu WH, Zhu RF, Lu YP. Ultrasonic enhancing amorphization during synthesis of calcium phosphate. Ultrason Sonochem 2014;21:499–504. https://doi.org/10.1016/J.ULTSONCH.2013.08.011.

[7] Vecstaudza J, Locs J. Novel preparation route of stable amorphous calcium phosphate nanoparticles with high specific surface area. J Alloys Compd 2017;700:215–22. https://doi.org/10.1016/J.JALLCOM.2017.01.038.

[8] Cuneyt Tas A. Submicron spheres of amorphous calcium phosphate forming in a stirred SBF solution at 55 °C. J Non Cryst Solids 2014;400:27–32. https://doi.org/10.1016/J.JNONCRYSOL.2014.04.031.

[9] Lee D, Kumta PN. Chemical synthesis and characterization of magnesium substituted amorphous calcium phosphate (MG-ACP). Mater Sci Eng C 2010;30:1313–7. https://doi.org/10.1016/J.MSEC.2010.05.009.

[10] Shahrezaee M, Raz M, Shishehbor S, Moztarzadeh F, Baghbani F, Sadeghi A, et al. Synthesis of Magnesium Doped Amorphous Calcium Phosphate as a Bioceramic for Biomedical Application: In Vitro Study. Silicon 2018;10:1171–9. https://doi.org/10.1007/S12633-017-9589-Y.

[11] Babaie E, Zhou H, Lin B, Bhaduri SB. Influence of ethanol content in the precipitation medium on the composition, structure and reactivity of magnesium-calcium

phosphate. Mater Sci Eng C 2015;53:204–11. https://doi.org/10.1016/J.MSEC.2015.04.011.

[12] Müller WEG, Ackermann M, Al-Nawas B, Righesso LAR, Muñoz-Espí R, Tolba E, et al. Amplified morphogenetic and bone forming activity of amorphous versus crystalline calcium phosphate/polyphosphate. Acta Biomater 2020;118:233–47. https://doi.org/10.1016/J.ACTBIO.2020.10.023.

[13] Jin L, Na LH, Liu FT, Gong LY, Lou DW, Zhang JP. Synthesis and adsorption properties for CdTe quantum dots of zero-valent iron/amorphous calcium phosphate composites. Chinese J Inorg Chem 2016;32:2025–33. https://doi.org/10.11862/CJIC.2016.251.

[14] Karimi M, Hesaraki S, Alizadeh M, Kazemzadeh A. A facile and sustainable method based on deep eutectic solvents toward synthesis of amorphous calcium phosphate nanoparticles: The effect of using various solvents and precursors on physical characteristics. J Non Cryst Solids 2016;443:59–64. https://doi.org/10.1016/J.JNONCRYSOL.2016.04.026.

[15] Rabadjieva D, Gergulova R, Titorenkova R, Tepavitcharova S, Dyulgerova E, Balarew C, et al. Biomimetic transformations of amorphous calcium phosphate: kinetic and thermodynamic studies. J Mater Sci Mater Med 2010;21:2501–9. https://doi.org/10.1007/S10856-010-4103-8.

[16] Hoeher AJ, Mergelsberg ST, Borkiewicz OJ, Michel FM. Impacts of initial Ca/P on amorphous calcium phosphate. Cryst Growth Des 2021;21:3736–45. https://doi.org/10.1021/ACS.CGD.1C00058/ASSET/IMAGES/LARGE/CG1C00058_0007.JPEG.

[17] Rabadjieva D, Tepavitcharova S, Sezanova K, Gergulova R. Chemical Equilibria Modeling of Calcium Phosphate Precipitation and Transformation in Simulated Physiological Solutions. J Solution Chem 2016;45:1620–33. https://doi.org/10.1007/S10953-016-0528-0.

[18] Erceg I, Selmani A da, Gajović A, Radatović B, Šegota S, Ćurlin M, et al. Precipitation at room temperature as a fast and versatile method for calcium phosphate/tio2 nanocomposites synthesis. Nanomaterials 2021;11. https://doi.org/10.3390/NANO11061523/S1.

[19] Chaudhry AA, Khalid H, Zahid M, Ijaz K, Akhtar H, Younas B, et al. Zinc containing calcium phosphates obtained via microwave irradiation of suspensions. Mater Chem Phys 2022;276:124921. https://doi.org/10.1016/J.MATCHEMPHYS.2021.124921.

[20] Yu T, Gao C, Ye J, Zhang M. Synthesis and Characterization of a Novel Silver-Substituted Calcium Phosphate Cement. J Mater Sci Technol 2014;30:686–91. https://doi.org/10.1016/J.JMST.2014.03.005.

[21] Erceg I, Maltar-Strmečki N, Domazet Jurašin D, Strasser V, Curlin M, Lyons DM, et al. Comparison of the Effect of the Amino Acids on Spontaneous Formation and

Transformation of Calcium Phosphates. Cryst 2021, Vol 11, Page 792 2021;11:792. https://doi.org/10.3390/CRYST11070792.

[22] Zyman ZZ, Rokhmistrov D V., Glushko VI. Structural and compositional features of amorphous calcium phosphate at the early stage of precipitation. J Mater Sci Mater Med 2010;21:123–30. https://doi.org/10.1007/S10856-009-3856-4.

[23] Leeuwenburgh SCG, Wolke JGC, Siebers MC, Schoonman J, Jansen JA. In vitro and in vivo reactivity of porous, electrosprayed calcium phosphate coatings. Biomaterials 2006;27:3368–78. https://doi.org/10.1016/J.BIOMATERIALS.2006.01.052.

[24] Luo H, Xiong G, Wan Y. In situ phosphorus K-edge X-ray absorption spectroscopy studies of calcium-phosphate formation and transformation on the surface of bacterial cellulose nanofibers. Cellulose 2014;21:3303–9. https://doi.org/10.1007/S10570-014-0359-3/FIGURES/4.

[25] Gong CY, Geng ZG, Dong A Le, Ye XX, Wang GZ, Zhang YX. Highly Efficient and Selective Removal of Pb(II) ions by Sulfur-Containing Calcium Phosphate Nanoparticles. Chinese J Chem Phys 2016;29:607. https://doi.org/10.1063/1674-0068/29/CJCP1603045.

[26] Guerra-López J, González R, Gómez A, Pomés R, Punte G, Della Védova CO. Effects of Nickel on Calcium Phosphate Formation. J Solid State Chem 2000;151:163–9. https://doi.org/10.1006/JSSC.1999.8615.

[27] Borovik P, Oestreicher V, Huck-Iriart C, Jobbágy M. Amorphous Calcium Phosphates: Solvent-Controlled Growth and Stabilization through the Epoxide Route. Chem – A Eur J 2021;27:10077–86. https://doi.org/10.1002/CHEM.202005483.

[28] Gelli R, Briccolani-Bandini L, Pagliai M, Cardini G, Ridi F, Baglioni P. Exploring the effect of Mg2+ substitution on amorphous calcium phosphate nanoparticles. J Colloid Interface Sci 2022;606:444–53. https://doi.org/10.1016/J.JCIS.2021.08.033.

[29] Gang X, Wang L, Jia J, Wang H, Lian X, Gao X, et al. Synthesis and biological evaluation of fluorescent hyaluronic acid modified amorphous calcium phosphate drug carriers for tumor-targeting. Int J Biol Macromol 2021;182:1445–54. https://doi.org/10.1016/J.IJBIOMAC.2021.05.068.

[30] Wang XM, Yan Y, Ren HH, Li SY. Nano-amorphous calcium phosphate doped with citrate: Fabrication, structure, and evaluation of the biological performance. Https://DoiOrg/101177/0885328219845969 2019;34:273–83. https://doi.org/10.1177/0885328219845969.

[31] Wang X, Zhao D, Ren H, Yan Y, Li S. Biological evaluation of the modified nano-amorphous phosphate calcium doped with citrate/poly-amino acid composite as a potential candidate for bone repair and reconstruction. J Mater Sci Mater Med 2021;32:1–18. https://doi.org/10.1007/S10856-020-06482-7/TABLES/2.

[32] Ortiz-Gómez I, Ramírez-Rodríguez GB, Capitán-Vallvey LF, Salinas-Castillo A, Delgado-López JM. Highly stable luminescent europium-doped calcium phosphate nanoparticles for creatinine quantification. Colloids Surfaces B Biointerfaces 2020;196:111337. https://doi.org/10.1016/J.COLSURFB.2020.111337.

[33] Tas AC. X-ray-amorphous calcium phosphate (ACP) synthesis in a simple biomineralization medium. J Mater Chem B 2013;1:4511–20. https://doi.org/10.1039/C3TB20854K.

[34] Rodrigues A, Lebugle A. Influence of ethanol in the precipitation medium on the composition, structure and reactivity of tricalcium phosphate. Colloids Surfaces A Physicochem Eng Asp 1998;145:191–204. https://doi.org/10.1016/S0927-7757(98)00660-8.

[35] Le Grill S, Soulie J, Coppel Y, Roblin P, Lecante P, Marsan O, et al. Spray-drying-derived amorphous calcium phosphate: a multi-scale characterization. J Mater Sci 2020 562 2020;56:1189–202. https://doi.org/10.1007/S10853-020-05396-7.

[36] Bradt JH, Mertig M, Teresiak A, Pompe W. Biomimetic Mineralization of Collagen by Combined Fibril Assembly and Calcium Phosphate Formation. Chem Mater 1999;11:2694–701. https://doi.org/10.1021/CM991002P.

[37] Hakimimehr D, Liu DM, Troczynski T. In-situ preparation of poly(propylene fumarate)—hydroxyapatite composite. Biomaterials 2005;26:7297–303. https://doi.org/10.1016/J.BIOMATERIALS.2005.05.065.

[38] Tao J, Pan H, Zhai H, Wang J, Li L, Wu J, et al. Controls of tricalcium phosphate single-crystal formation from its amorphous precursor by interfacial energy. Cryst Growth Des 2009;9:3154–60. https://doi.org/10.1021/CG801130W/SUPPL_FILE/CG801130W_SI_001.PDF.

[39] Karimi M, Hesaraki S, Alizadeh M, Kazemzadeh A. Time and temperature mediated evolution of CDHA from ACP nanoparticles in deep eutectic solvents: Kinetic and thermodynamic considerations. Mater Des 2017;122:1–10. https://doi.org/10.1016/J.MATDES.2017.02.076.

[40] Gutiérrez MC, Jobbágy M, Ferrer ML, Del Monte F. Enzymatic synthesis of amorphous calcium phosphate-chitosan nanocomposites and their processing into hierarchical structures. Chem Mater 2008;20:11–3. https://doi.org/10.1021/CM7020164/SUPPL_FILE/CM7020164.PDF.

[41] Sinusaite L, Renner AM, Schütz MB, Antuzevics A, Rogulis U, Grigoraviciute-Puroniene I, et al. Effect of Mn doping on the low-temperature synthesis of tricalcium phosphate (TCP) polymorphs. J Eur Ceram Soc 2019;39:3257–63. https://doi.org/10.1016/J.JEURCERAMSOC.2019.03.057.

[42] Salahi E, Heinrich JG. Synthesis and thermal behaviour of β tricalcium phosphate precipitated from aqueous solutions. Http://DxDoiOrg/101179/096797803225001597

2013;102:79–83. https://doi.org/10.1179/096797803225001597.

[43] Bucur AI, Bucur R, Vlase T, Doca N. Thermal analysis and high-temperature X-ray diffraction of nano-tricalcium phosphate crystallization. J Therm Anal Calorim 2011 1071 2011;107:249–55. https://doi.org/10.1007/S10973-011-1753-9.

[44] Garskaite E, Gross KA, Yang SW, Yang TCK, Yang JC, Kareiva A. Effect of processing conditions on the crystallinity and structure of carbonated calcium hydroxyapatite (CHAp). CrystEngComm 2014;16:3950–9. https://doi.org/10.1039/C4CE00119B.

[45] Arifuzzaman SM, Rohani S. Experimental study of brushite precipitation. J Cryst Growth 2004;267:624–34. https://doi.org/10.1016/J.JCRYSGRO.2004.04.024.

[46] Yan D, Lou Y, Han Y, Wickramaratne MN, Dai H, Wang X. Controllable synthesis of poly(acrylic acid)-stabilized nano-hydroxyapatite suspension by an ultrasound-assisted precipitation method. Mater Lett 2018;227:9–12. https://doi.org/10.1016/J.MATLET.2018.04.124.

[47] Seckler MM, Danese M, Derenzo S, Valarelli J V, Giulietti M, Rodríguez-Clemente R. Influence of Process Conditions on Hydroxyapatite Crystallinity Obtained by Direct Crystallization 1999.
